# Predictive Value of Novel Inflammation-Based Biomarkers for Pulmonary Hypertension in the Acute Exacerbation of Chronic Obstructive Pulmonary Disease

**DOI:** 10.1155/2019/5189165

**Published:** 2019-10-14

**Authors:** Huanhuan Zuo, Xiaochen Xie, Jiahuan Peng, Lixin Wang, Rong Zhu

**Affiliations:** ^1^Department of Respiratory Medicine, The Huaian Clinical College of Xuzhou Medical University, Huaian 223001, China; ^2^Department of Biostatistics School of Public Health, Nanjing Medical University, Nanjing 210000, China

## Abstract

Recently, there has been an increasing interest in the potential clinical use of several inflammatory indexes, namely, neutrophil-to-lymphocyte ratio (NLR), platelet-to-lymphocyte ratio (PLR), and systemic-immune-inflammation index (SII). This study aimed at assessing whether these markers could be early indicators of pulmonary hypertension (PH) in patients with acute exacerbation of chronic obstructive pulmonary disease (AECOPD). A total of 185 patients were enrolled in our retrospective study from January 2017 to January 2019. Receiver operating characteristic curve (ROC) and area under the curve (AUC) were used to evaluate the clinical significance of these biomarkers to predict PH in patients with AECOPD. According to the diagnostic criterion for PH by Doppler echocardiography, the patients were stratified into two groups. The study group consisted of 101 patients complicated with PH, and the control group had 84 patients. The NLR, PLR, and SII values of the PH group were significantly higher than those of the AECOPD one (*p* < 0.05). The blood biomarker levels were positively correlated with NT-proBNP levels, while they had no significant correlation with the estimated pulmonary arterial systolic pressure (PASP) other than PLR. NLR, PLR, and SII values were all associated with PH (*p* < 0.05) in the univariate analysis, but not in the multivariate analysis. The AUC of NLR used for predicting PH was 0.701 and was higher than PLR and SII. Using 4.659 as the cut-off value of NLR, the sensitivity was 81.2%, and the specificity was 59.5%. In conclusion, these simple markers may be useful in the prediction of PH in patients with AECOPD.

## 1. Introduction

Chronic obstructive pulmonary disease (COPD), characterized by an incompletely reversible airflow limitation, is not just a chronic inflammatory response involving the airways but a systemic chronic inflammatory syndrome. It is a worldwide health-care burden which poses a significant public health challenge [1]. The Global Burden of Disease Study estimated that there were 174.5 million prevalent COPD patients worldwide in 2015 [2], and COPD will represent the third leading cause of death globally by 2030 [3]. AECOPD indicates a prolonged (≥48 h) worsening of a patient's clinical respiratory manifestations that require additional medications or are severe enough to warrant hospital admission [4]. It is a complex and life-threatening condition which is responsible for a growing mortality, a large proportion of health-care expenditure, an increased risk of dying, and the development of complications in the progression of the disease [5].

Pulmonary hypertension (PH) is a severe and poor prognosis complication of COPD. Although the primary disease progresses slowly, once combined with PH the symptoms aggravate, mortality surges, and the risk of AECOPD increases. COPD patients with PH have a poor long-term prognosis with a median postdiagnosis survival of only 2 to 5 years [6]. Early diagnosis and timely treatment are particularly important in the course of disease progression in our clinical work. The detection methods for PH are mainly divided into invasive and noninvasive examinations. Although right heart catheterization is the “gold standard” for the diagnosis of PH, it is relatively complicated, expensive, and invasive. As a result, Doppler echocardiography is recommended by the ESC/ERS Guidelines as the primary noninvasive diagnostic instrument in suspected pulmonary arterial hypertension (PAH) in COPD patients [7].

However, the prediction of PH appears to be an impossible mission especially in some community hospitals with inferior methods of examination. Thus, a growing number of researchers are extensively focusing on finding a noninvasive and more easily obtainable biomarker that enables stratification of PH in COPD patients. Recently, NLR, PLR, or SII have been associated with inflammation-linked diseases (malignancy [8], ulcerative colitis [9], and ANCA-associated vasculitis [10], for example). However, as far as we know, few studies have evaluated the utility of these blood-based molecules as predictive biomarkers of PH in AECOPD patients. This article will summarize the predictive significance of these various inflammatory indices and estimate the independent risk factors correlated with PH.

## 2. Methods

### 2.1. Study Population

Patients diagnosed with AECOPD (*n* = 185) were registered in this retrospective study. All patients evaluated for PH in our study underwent Doppler echocardiography and were divided into study and control groups depending on whether they also had PH. 101 AECOPD patients with PH were included in the study group, and the remaining eighty-four patients were assigned to the control group.

The inclusion criteria are as follows: (1) age ≥ 40 years; (2) a COPD diagnosis supported by pulmonary function tests of airflow obstruction even with a bronchodilator (forced expiratory volume in 1 second (FEV1)/forced vital capacity (FVC) < 70%) when clinically stable for at least 3 months; (3) a primary diagnosis of AECOPD, defined as a deterioration of respiratory symptoms, such as dyspnea sensation, coughing, or purulent sputum that is beyond normal variability and severe enough to result in hospitalization [11]; and (4) meeting the diagnostic criteria for PH according to the 2015 European Society of Cardiology and the European Respiratory Society (ESC/ERS) Guidelines for the Diagnosis and Treatment of Pulmonary Hypertension Pressure diagnostic criteria [7], both of whom consider the diagnostic criteria for PH by echocardiography as follows: mild PH—36 mmHg ≤ PASP ≤ 50 mmHg; moderate PH—51 mmHg ≤ PASP ≤ 70 mmHg; and severe PH—PASP > 70 mmHg.

The exclusion criteria includes the following: (1) pregnant and lactating women; (2) idiopathic pulmonary hypertension; (3) other causes of pulmonary arterial hypertension (PAH), such as interstitial lung disease, congenital heart disease, heart valve disease, and acute left heart dysfunction; (4) suffering from other systemic diseases, such as left heart disease, autoimmune disease, blood system disease, thromboembolism disease, malignancy, and acute infectious diseases; and (5) patients who recently received a blood transfusion.

Our study protocol was approved by the ethics committee of Jiangsu Province Huaian No. 1 People's Hospital and was in agreement with the guidelines of the Declaration of Helsinki. An informed consent was not signed by each patient because of the retrospective design of this study.

### 2.2. Data Collection

The following clinical pathological data were obtained by reviewing the patients' medical records: age, gender, body mass index (BMI), smoking index, hospital stay duration, the course of the disease, underlying disease, and laboratory results during the first 12 hours after admission to the hospital. BMI is defined as a person's weight in kilograms divided by the square of the height in meters (kg/m^2^). The definition of the smoking index is the average root number per day multiplied by years of smoking.

Inflammatory indices were calculated as follows: NLR = neutrophil counts/lymphocyte counts, PLR = platelet counts/lymphocyte counts, and SII = platelet counts × neutrophil counts/lymphocyte counts.

### 2.3. Statistical Analysis

All statistical analyses were performed using the Statistical Analysis System version 9.4 (SAS Institute, Cary, NC, USA). The Shapiro-Wilk method was used to test the normality of the data. Normally distributed numerical variables were presented as mean ± standard deviation, and the parameters which showed a nonnormal distribution were presented as median-interquartile range. Categorical variables were presented as frequencies and percentages. Normally distributed numerical variables were compared using the unpaired Student *t*-test. A Wilcoxon signed-rank test was used for the comparison of nonnormally distributed numerical variables which did not show a normal distribution after logarithmic transformation. Comparison of more than two independent groups was performed using the ANOVA and the Kruskal-Wallis test according to the distribution state. Differences between categorical variables were analyzed using a Pearson chi-square test. The correlation coefficients and significance of the continuous variables were assessed using a Spearman correlation test. Independent risk factors were analyzed by univariate and multivariate logistic regression. The Youden index method with a receiver operating characteristic (ROC) curve analysis was used to determine the optimal cut-off values of the predictive parameters of PH. The predictive probabilities were compared using the corresponding areas under the curve (AUCs) with 95% confidence intervals (CI). A value of *p* < 0.05 was considered statistically significant.

## 3. Results

### 3.1. Subjects at Baseline

We retrospectively enrolled a total of 185 patients (age: 71.18 ± 8.17) with a diagnosis of AECOPD who met the inclusion criteria, including 141 males and 44 females (male proportion: 76.22%). 101 patients with PH secondary to COPD were included in the study group. PH was mild in 50 (49.50%) patients, moderate in 33 (32.67%), and severe in 18 (17.82%) patients in the study group. Baseline demographic characteristics and clinical data of the subjects reviewed are summarized in Table 1. The mean age and gender did not differ significantly between the study group and the control one (age: 72.06 ± 7.90 versus 70.12 ± 8.41, *p* = 0.108; male proportion: 76.24% versus 76.19%, *p* = 0.994). Confounding factors were compared, including the smoking index, BMI, hospital stays, and underlying disease. We did not find any differences in terms of BMI and smoking index between the two groups (all *p* > 0.05). Length of hospital stay, course of the disease, and coexisting illnesses (hypertension or diabetes) were not significantly different in patients with an exacerbation of COPD compared with those with PH. There was no difference in the demographic characteristics between the two groups, nor did they differ in confounding factors and comorbidities (*p* > 0.05). Therefore, the laboratory parameters were comparable.

### 3.2. Overall Comparison of the Laboratory Parameters and Baseline Echocardiographic Variables between the Study Group and the Control Group

The lymphocyte count was significantly decreased in the study group compared to the control one (0.91 versus 1.24, *p* ≤ 0.001), but no significant differences among white blood cells, red blood cells, hemoglobin, neutrophils, platelets, and monocytes were presented between the two groups (*p* > 0.05) (Table 2).

As for the inflammatory indexes, patients with PH had a significantly higher median NLR value (6.52 versus 4.08, *p* ≤ 0.001), higher median PLR value (220.88 versus 156.71, *p* ≤ 0.001), and higher median SII value (1453.38 versus 884.87, *p* ≤ 0.001) than the AECOPD group. Among the biochemical parameters, the NT-proBNP and albumin levels in the study group were significantly higher compared to those in the control one (653.00 versus 133.00, *p* ≤ 0.001; 36.51 ± 4.75 versus 38.44 ± 3.78, *p* = 0.003). Furthermore, we found that the PaCO_2_ value in the AECOPD group complicated by PH was higher compared with that in the AECOPD controls, 50.10 and 44.35, respectively (*p* = 0.002). Compared with the AECOPD group, the HCO_3_^−^ value of the PH one was higher, 31.50 and 28.60, respectively (*p* = 0.002). The Lac of the study group was significantly higher than that of patients with COPD exacerbation (1.60 versus 1.50, *p* = 0.032).

Comparison of the D-Dimer levels of the two groups revealed that this value (0.65 versus 0.39, *p* ≤ 0.001) was increased in the PH group compared to the AECOPD one. However, fibrinogen was similar in both groups (4.26 versus 4.30, *p* = 0.708). The estimated hemodynamic parameters by Doppler echocardiography of the two groups were also listed in Table 2. The right atrium diameter (RAD) and right ventricular diameter (RVD) were significantly higher in the study group compared with those in the control one (34.38 ± 6.60 versus 30.74 ± 3.80, *p* ≤ 0.001; 18 versus 17, *p* = 0.020). The left atrium diameter (LAD), left ventricular end diastolic diameter (LVDD), and left ventricular ejection fraction (LVEF) of the two groups were not significantly different (*p* > 0.05).

To evaluate the association between inflammatory indexes and PH, we further compared the levels of NLR, PLR, and SII in patients categorized by PH severity. Patients with severe PH had a higher PLR than those with mild and moderate PH. PLR and *p* values for mild and moderate groups in comparison with the severe PH group (326.59) were as follows: mild PH, 210.64 (*p* = 0.013) and moderate PH, 210.31 (*p* = 0.021). As for NLR and SII, no significant differences were observed between either the mild or the moderate PH groups and the severe group. The Doppler echocardiography parameters of the PH group are listed in Table 3. LAD, LVDD, and LVEF of the three groups were not significantly different. PTRV and PASP were significantly higher in the severe group compared with the moderate and mild ones (4.31 versus 3.47 versus 2.90, *p* ≤ 0.001; 79.50 ± 5.34 versus 58.18 ± 5.41 versus 42.98 ± 3.94, *p* ≤ 0.001).

### 3.3. Association of the Comparable Data with the Estimated PASP and the NT-proBNP

The relationship between the estimated PASP (or NT-proBNP) and the laboratory parameters is shown in Table 4.

The laboratory parameters with differences between the two groups were further included in the correlation analysis with the estimated PASP and the NT-proBNP, including lymphocytes, NLR, PLR, SII, NT-proBNP, PaCO_2_, HCO_3_^−^, Lac, and D-Dimer. According to the Spearman correlation analysis, the estimated PASP was associated with NT-proBNP (*r* = 0.500, *p* < 0.001). There was a significant but weak correlation of PASP with lymphocytes (*r* = −0.265, *p* = 0.007), PLR (*r* = 0.235, *p* = 0.018), PaCO_2_ (*r* = 0.403, *p* < 0.001), HCO_3_^−^ (*r* = 0.427, *p* < 0.001), and D-Dimer (*r* = 0.220, *p* = 0.027), while there was no significant correlation with NLR, SII, and Lac. NT-proBNP showed a negative correlation with lymphocytes (*r* = −0.386, *p* < 0.001), and a positive correlation with NLR (*r* = 0.340, *p* < 0.001), PLR (*r* = 0.355, *p* < 0.001), SII (*r* = 0.288, *p* < 0.001), PaCO_2_ (*r* = 0.268, *p* < 0.001), HCO_3_^−^ (*r* = 0.280, *p* < 0.001), and D-Dimer (*r* = 0.318, *p* < 0.001).

### 3.4. Univariate and Multivariate Analysis of the Occurrence of Pulmonary Hypertension

The variables that were significantly different between the two groups were also tested in the univariate analysis. This analysis revealed that the factors impacting PH were lymphocytes, NLR, PLR, SII, NT-proBNP, PaCO_2_, HCO_3_^−^, Lac, and D-Dimer (Table 5). The parameters identified as potential risk markers in the univariate analysis were further included in the multivariate logistic regression model (*p* < 0.05). Multivariate analyses identified NT-proBNP (OR: 1.003; 95% confidence interval (CI): 1.001-1.005; *p* < 0.001) as the independent risk factor correlated with PH. Nevertheless, NLR, PLR, and SII did not remain as independent predictors of PH.

### 3.5. Comparative Analysis of the Discriminative Ability of the Inflammatory Markers and NT-proBNP

A receiver operating characteristic curve (ROC) was generated to predict PH in AECOPD patients. The predictive accuracy values of the inflammatory markers and NT-proBNP are listed in Table 6.

**Figure 1:**
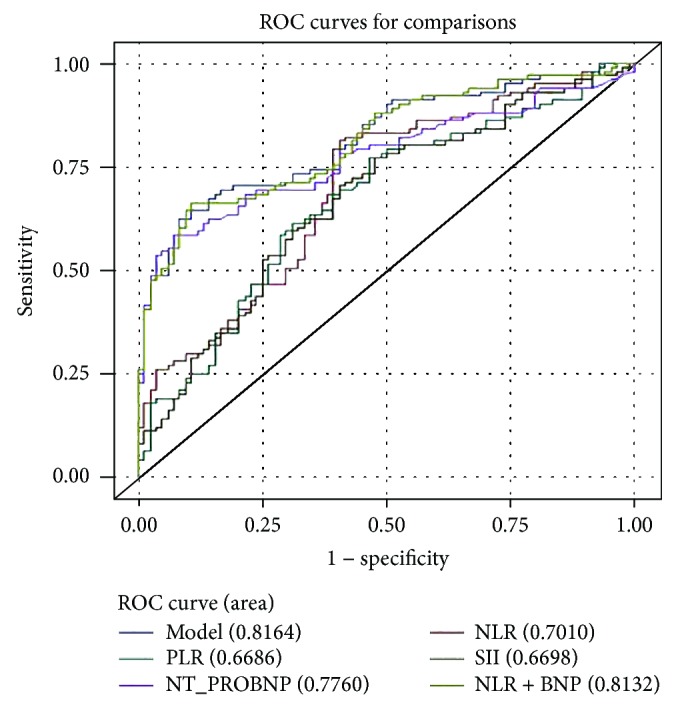
ROC curves for determining the cut-off value of NLR, PLR, SII, and NT-proBNP for predicting PH in AECOPD patients. Abbreviations: NLR—neutrophil-to-lymphocyte ratio; PLR—platelet-to-lymphocyte ratio; SII—systemic-immune-inflammation index.

Of the novel inflammatory markers, the NLR AUC (0.701; 95% confidence interval (CI), 0.629–0.766) was greater than that of PLR (AUC, 0.669; 95% CI, 0.596–0.736) and SII (AUC, 0.670; 95% CI, 0.597–0.737). The optimal cut-off value of NLR for predicting PH was 4.659, which yielded a 81.2% sensitivity and a 59.5% specificity. An SII of 1012 was considered the optimal cut-off value and the sensitivity and specificity were 70.3% and 59.5%, respectively. Using a PLR cut-off value of 160.0, the sensitivity and specificity for PH were 77.2% and 53.6%, respectively. The optimal cut-off value for NT-proBNP was 384.0 with a 58.4% sensitivity and a 92.9% specificity (AUC = 0.776). In order to improve the diagnostic efficacy of COPD-related pulmonary hypertension, we further examined the feasibility of the combined prediction of NLR and NT-proBNP. The prediction accuracy of NLR combined with NT-proBNP (AUC = 0.813) was higher than that of NLR or NT-proBNP alone.  Figure 1 shows the ROC curves of the predictive parameters of PH in patients with AECOPD.

## 4. Discussion

This study showed that NLR, PLR, and SII were significantly higher in PH patients secondary to COPD than in the AECOPD controls. In addition, these markers can be used to predict PH in AECOPD patients. In these cases, NLR has been shown to be superior to PLR and SII in its discriminative ability.

PH induced by COPD can lead to increased pulmonary arterial pressure, elevated pulmonary vascular resistance, and progressive right heart failure, which results from increasing right ventricular afterload. The progress of PH is associated with a significant increase in clinical deterioration and risk of death. The pathogenesis of PH is due to the maladaptation of various vasomotor factors secreted by injured endothelial cells, resulting in early pulmonary vasoconstriction and later pulmonary vascular remodeling. Increasing evidence suggests that inflammation plays an extremely decisive role in the progression of PH [12]. The pathophysiology of pulmonary vascular remodeling in PH is not only the pathological damage of endothelial cell function but also the excessive perivascular infiltration of inflammatory cells [13].

Lymphocytes decline in autoimmune diseases and are responsible for peripheral immune tolerance. Consistent with previously published literature [14, 15], the current study showed that lymphocyte counts in PH patients were significantly lower compared with those in the control AECOPD group, which might be able to reflect the balance between host inflammatory status and immune status. The classification of T lymphocytes in PH patients is obviously different from that of the healthy population. Studies on the lymphocyte subsets in patients with PH are controversial. Stacher et al. [16] discovered that in different types of pulmonary hypertension, almost all of them were accompanied by a large number of inflammatory cells (mainly lymphocytes) infiltrating into the lung perivascular region and the interstitium. Another study showed that CD8^+^ cytotoxic T cells were reduced and regulatory T cells were increased in patients with idiopathic pulmonary hypertension [17]. Furthermore, researchers have found that the level of Th17 cells and interleukin-17A (IL-17A) increased in PH patients associated with connective tissue disease [18] and idiopathic pulmonary hypertension (IPH) [19], which suggested that Th17 cells may play a crucial role in promoting the development of PH. An upregulation of CD25^+^Foxp3^+^ cells in CD8^+^ T cells and a downregulation of CD4^+^CD25^+^Foxp3^+^ T cells were also observed in PAH patients compared to healthy controls by Zhu et al. [20].

There was no significant difference of blood neutrophil level between the non-PH group and the PH group in AECOPD patients in our study. However, neutrophil infiltration has been observed in murine lungs in hypoxia-induced PH mice [21], and the role of neutrophils in the pathogenesis of PH was not fully understood. A study demonstrated that circulating inflammatory mediators have been associated with poor clinical outcomes in PH [22]. Neutrophils release a consistent amount of reactive oxygen species (ROS) and further trigger massive amplification of the inflammatory cascade reaction by activating mitogen-activated protein kinase (MAPK) and redox-sensitive transcription factors [23]. IL-6, secreted by neutrophils, promotes pulmonary artery smooth muscle cell (PASMC) proliferation by upregulating the expression of vascular endothelial growth factor (VEGF) and downregulating the expression of pulmonary bone morphogenetic protein receptor type 2 (BMPR2) [24]. Soon et al. [25] observed that IL-6, IL-8, TNF-*α*, and other inflammatory factors were significantly higher during the development of PH than in the normal population. There are several reasons that can explain our results. Firstly, the sample size was small and may have affected the research result. Secondly, the treatment received with corticosteroids before admission may have affected the white blood cell counts [26]. Thirdly, the patients in this study were older and may have been less responsiveness to inflammation.

In this study, NLR, PLR, and SII were all significantly higher and the result was consistent with established associations between PH and host immune and inflammatory environments. NLR, based on neutrophil count and lymphocyte count, has been increasingly investigated as a marker of systemic inflammation, especially because it is a relatively inexpensive and widely available evaluation tool. Recently, NLR has been extensively studied in COPD. Several studies have shown that NLR was linked with disease severity and may be useful in the prediction of the prognosis of COPD. Gunay et al. [27] found that compared with stable COPD patients (NLR = 2.59), the NLR value of the AECOPD group was significantly increased (NLR = 4.28), and the NLR value of COPD patients was significantly higher than that of the healthy control group (NLR = 1.71). Yao et al. [28] discovered that higher levels of NLR (>6.24) and PLR (>182.68) predicted an increased risk of hospital mortality in the patients with AECOPD. For the first time, a study demonstrated a significant increase in NLR values in patients with PAH compared with healthy volunteers [14]. Özpelit et al. subsequently reported that NLR may be directly related to the severity and prognosis of PAH [15]. Nevertheless, few studies have concentrated on the predictive ability of NLR in PH patients induced by COPD. In this study, the level of NLR was significantly higher in PH patients compared with AECOPD patients. The ROC curve analysis showed that the AUC of the NLR for predicting PH was greater than that of PLR and SII, and the predictive ability of the NT-proBNP was stronger than NLR. However, for some community hospitals with backward medical facilities, NLR is easy to calculate from a routine complete blood count without increasing the patients' burden and is considerably cheaper than NT-proBNP. Use of NLR for predicting PH resulted in a greater sensitivity than for NT-proBNP (81.2% versus 58.4%), but NT-proBNP had a higher associated specificity of 92.9% in this cohort. The combination of NLR and NT-proBNP resulted in an AUC of 0.813. Thus, we can infer that NLR may be a more objective indicator of the balance between host inflammatory and immune responses than indicators such as PLR or SII.

To our knowledge, PLR and SII have not been studied in PH patients induced by COPD until now. We discovered that PLR and SII increased significantly in patients complicated with PH than in the AECOPD group. COPD patients have a hypercoagulable state due to long-term bed rest, hemodynamic abnormalities, and the hypoxia of cells. The platelet-related index can effectively evaluate the severity of COPD. PLR, based on platelet and lymphocyte count, was increased in AECOPD patients than in COPD and healthy controls and has been proven to be linked with poor prognosis in COPD patients [29]. The systemic-immune-inflammation index (SII), based on lymphocyte count, neutrophil count, and platelet count, is a comprehensive indicator with an important prognostic value for colorectal cancer [30], resectable pancreatic cancer [31], gastric cancer [32], and so on. Few studies have been concerned with the association between the novel inflammation-based biomarkers and the severity of PH in AECOPD combined with PH patients. We further evaluated the relationship between these biomarkers and the estimated PASP. As a result, these markers have no significant correlation with estimated PASP other than PLR, but were significantly correlated with NT-proBNP, a well-known factor that can predict disease progression in PH patients. From this, we can conclude that NLR and SII can be used for the early prediction of patients with PH, but have no statistically significant correlation with the severity of PH.

Blood gas parameters were also compared. Owing to some patients needing oxygen intake or invasive mechanical ventilation for a long time after admission, the partial pressure of arterial oxygen in the blood gas analysis was disturbed. Therefore, PH, PaCO_2_, and HCO_3_^−^ were utilized in our study. The PaCO_2_, HCO_3_^−^, and Lac values of the PH group were higher than those of the control one. Spearman's correlation analysis showed that the estimated PASP was positively correlated with PaCO_2_ and HCO_3_^−^. These results suggested that PaCO_2_ and HCO_3_^−^ may be related to the severity of pulmonary artery pressure, in addition to NLR or SII. In accordance with this, Samareh conducted a cross-sectional study of 1078 patients with severe PH in COPD [33]. This study illustrated that various factors, such as hypoxia and hypopnea, play a major role in the severity of PH in these patients. Under the influence of hypoxemia and hypercapnia, pulmonary vascular resistance is significantly increased due to pulmonary vasoconstriction or even vasospasm. As the disease progresses, pulmonary vascular remodeling eventually leads to PH.

## 5. Strengths and Limitations of This Study

There are some strengths and limitations to our study. First, this article maybe one of the few researches investigating NLR, PLR, and SII as novel inflammation-based biomarkers in patients with PH secondary to COPD. These markers can be regarded as a promising and convenient tool to predict PH in COPD patients. Second, some studies indicated that NLR is influenced by age and BMI [34, 35]. Therefore, in the clinical use of these indicators, it is still necessary to comprehensively consider the patient's age, medical history, BMI, etc. Our matching process adequately controlled for the potential confounders to make these novel markers more reliable. The limitations are as follows: First, our study was a single-center one with a small sample size, which means that the study sample included patients who are cared for by a single tertiary medical center. In addition, considering the critical condition of part of the AECOPD patients, lung function tests were not performed for the sake of these patients' safety. Second, invasive examination would not be indicated and ethical for all admitted COPD patients, and the estimated PASP measured by Doppler echocardiography was only moderately correlated with the values conducted by right heart catheterization. Third, the symptoms and quality of life expressed as St. George's Respiratory Questionnaire (SGRQ), Modified British Medical Research Council (mMRC) Questionnaire, and COPD Assessment Test (CAT) scores and the history of previous deteriorations could not be obtained due to its retrospective design.

## 6. Conclusion and Future Directions

From this study, we concluded that NLR, PLR, and SII can be used as practical means for the prediction of PH especially in community hospitals with poor medical infrastructures and the accuracy of NLR was higher than that of PLR and SII. The threshold of NLR was 4.659 for the early differential screening between AECOPD patients complicated by PH and patients with AECOPD alone. Given the grave prognosis of PH, larger multicenter, well-designed, prospective clinical studies are warranted to validate the use of these promising biomarkers, which are routinely measured on admission and require no extra cost in clinical practices. Understanding the critical role of the inflammatory signaling pathway in the pathophysiological mechanisms of PH may also lead to potential therapeutic targets in the future.

## Figures and Tables

**Table 1:** Baseline characteristics and clinical data of the enrolled subjects.

Characteristics	AECOPD group (*n* = 84)	PH group (*n* = 101)	*p* value
Age (years)	70.12 ± 8.41	72.06 ± 7.90	0.108
Gender (male), (*n*, %)	64 (76.19)	77 (76.24)	0.994
Hospital stay (day)	9.00 (7.00-11.00)	9.00 (7.00-10.00)	0.720
Course of disease (year)	10.00 (10.00-20.00)	10.00 (10.00-20.00)	0.537
BMI (kg/m^2^)	23.68 ± 3.64	22.73 ± 3.99	0.095
Smoking index (year root)	600 (200-800)	600 (200-1000)	0.322
Hypertension (*n*, %)	35 (41.67)	36 (35.64)	0.402
Diabetes (*n*, %)	11 (13.10)	11 (10.89)	0.645
NYHA classification (*n*, %)			
I	29 (34.52)	7 (6.93)	
II	45 (53.57)	37 (36.63)	
III	10 (11.91)	48 (47.53)	
IV	0	9 (8.91)	

Abbreviations: AECOPD—acute exacerbation of chronic obstructive pulmonary disease; PH—pulmonary hypertension; BMI—body mass index; NYHA—New York Heart Association.

**Table 2:** Comparison of the laboratory parameters and echocardiographic variables between the two groups.

Parameters	AECOPD group (*n* = 84)	PH group (*n* = 101)	*p* value
WBC (×10^9^/l)	7.72 (5.83-9.99)	7.90 (6.79-10.41)	0.432
RBC (×10^12^/l)	4.58 ± 0.59	4.55 ± 0.68	0.751
Hemoglobin (g/l)	136.21 ± 16.90	134.75 ± 19.14	0.586
Neutrophils (×10^9^/l)	5.75 (4.18-7.89)	6.26 (4.85-8.17)	0.063
Lymphocytes (×10^9^/l)	1.24 (0.95-1.59)	0.91 (0.66-1.26)	*p* ≤ 0.001
Monocytes (×10^9^/l)	0.52 (0.38-0.68)	0.54 (0.41-0.72)	0.576
Platelets (×10^9^/l)	201.50 (165.00-252.50)	193.00 (154.00-229.00)	0.202
NLR	4.08 (2.89-7.26)	6.52 (4.95-12.28)	*p* ≤ 0.001
PLR	156.71 (123.50-227.21)	220.88 (161.08-290.91)	*p* ≤ 0.001
SII	884.87 (554.77-1453.34)	1453.38 (952.45-2441.84)	*p* ≤ 0.001
Albumin (g/l)	38.44 ± 3.78	36.51 ± 4.75	0.003
NT-proBNP (pg/ml)	133.00 (76.00-238.50)	653.00 (167.00-1565.00)	*p* ≤ 0.001
PH	7.41 ± 0.04	7.40 ± 0.05	0.080
PaCO_2_ (mmHg)	44.35 (40.90-50.00)	50.10 (42.30-61.90)	0.002
HCO_3_^−^ (mmol/l)	28.60 (26.90-31.20)	31.50 (27.30-37.40)	0.002
Lac (mmol/l)	1.50 (1.00-1.80)	1.60 (1.20-2.10)	0.032
D-Dimer (*μ*g/ml)	0.39 (0.28-0.60)	0.65 (0.37-1.38)	*p* ≤ 0.001
Fibrinogen (g/l)	4.30 (3.49-5.32)	4.26 (3.32-6.17)	0.708
LAD (mm)	27 (26-29)	29 (24-31.5)	0.217
LVDD (mm)	44.23 ± 4.41	43.40 ± 5.46	0.254
RAD (mm)	30.74 ± 3.80	34.38 ± 6.60	*p* ≤ 0.001
RVD (mm)	17 (16-18)	18 (17-20)	0.020
LVEF	68 (66-68)	68 (65-68)	0.296

Abbreviations: AECOPD—acute exacerbation of chronic obstructive pulmonary disease; PH—pulmonary hypertension; WBC—white blood cell; RBC—red blood cell; NLR—neutrophil-to-lymphocyte ratio; PLR—platelet-to-lymphocyte ratio; SII—systemic-immune-inflammation index; PaCO_2_—partial pressure of carbon dioxide; HCO_3_^−^—bicarbonate ion; Lac—lactic acid; LAD—left atrium diameter; LVDD—left ventricular end diastolic diameter; RAD—right atrium diameter; RVD—right ventricular diameter; LVEF—left ventricular ejection fraction.

**Table 3:** Laboratory parameters and echocardiographic variables based on severity of PH.

	Mild PH (*n* = 50)	Moderate PH (*n* = 33)	Severe PH (*n* = 18)	*p* value
Lym (×10^9^/l)	1.04 ± 0.43^b^	1.00 ± 0.47^c^	0.68 ± 0.37^b,c^	0.009
NLR	6.07 (4.86-11.25)	6.29 (5.05-10.62)	7.73 (4.80-17.73)	0.372
PLR	210.64 (153.61-277.05)^b^	210.31 (160.63-263.37)^c^	326.59 (232.77-443.02)^b,c^	0.010
SII	1473.25 (813.27-2448.08)	1299.09 (932.72-2352.89)	1611.04 (1047.50-2999.36)	0.432
Albumin (g/l)	37.85 (34.65-41)	36.10 (32.60-39.35)	35.40 (33.43-36.85)	0.158
NT-proBNP (pg/ml)	237.50 (108-1050.25)^a,b^	887 (274-3296)^a^	1588 (587-5296)^b^	*p* ≤ 0.001
PaCO_2_ (mmHg)	45.70 (39.25,51.38)^a^	60.10 (49.55-72.05)^a^	56.55 (40.85-63.78)	*p* ≤ 0.001
HCO_3_^−^ (mmol/l)	28.30 (26.70-32.20)^a,b^	36.30 (32-40.60)^a^	35.55 (28.13-39.43)^b^	*p* ≤ 0.001
Lac (mmol/l)	1.71 ± 0.57	1.58 ± 0.63	1.84 ± 0.90	0.389
D-Dimer (*μ*g/ml)	0.52 (0.37-0.94)	0.93 (0.39-2.30)	1.15 (0.38-1.73)	0.099
LAD (mm)	27.34 ± 5.14	29.09 ± 5.37	30.06 ± 5.18	0.114
LVDD (mm)	43 (40-47)	45 (41-47)	42 (35-46.25)	0.190
RAD (mm)	31.42 ± 5.28^a,b^	36.33 ± 5.53^a^	39 ± 7.90^b^	*p* ≤ 0.001
RVD (mm)	17 (16-18)^a,b^	19 (17-22)^a^	20.5 (17-32.5)^b^	*p* ≤ 0.001
PTRV (m/s)	2.9 (2.81-3.06)^a,b^	3.47 (3.33-3.64)^a,c^	4.31 (4.06-4.88)^b,c^	*p* ≤ 0.001
PASP (mmHg)	42.98 ± 3.94^a,b^	58.18 ± 5.41^a,c^	79.50 ± 5.34^b,c^	*p* ≤ 0.001
LVEF	68 (65-68)	67 (65-68)	66 (65-68)	0.254

Abbreviations: Lym—lymphocytes; NLR—neutrophil-to-lymphocyte ratio; PLR—platelet-to-lymphocyte ratio; SII—systemic-immune-inflammation index; PaCO_2_—partial pressure of carbon dioxide; HCO_3_^−^—bicarbonate ion; Lac—lactic acid; LAD—left atrium diameter; LVDD—left ventricular end diastolic diameter; RAD—right atrium diameter; RVD—right ventricular diameter; PTRV—peak tricuspid regurgitation velocity; PASP—pulmonary artery systolic pressure; LVEF—left ventricular ejection fraction. ^a^*p* < 0.05 for mild PH vs. moderate PH; ^b^*p* < 0.05 for mild PH vs. severe PH; ^c^*p* < 0.05 for moderate PH vs. severe PH.

**Table 4:** Relationship between the statistically different indicators and NT-proBNP (or PASP).

Parameters	NT-proBNP		PASP	
	*r* value	*p* value	*r* value	*p* value
Lymphocyte (10^9^/l)	-0.386	<0.001	-0.265	0.007
NLR	0.340	<0.001	0.087	0.389
PLR	0.355	<0.001	0.235	0.018
SII	0.288	<0.001	0.069	0.494
NT-proBNP (pg/ml)	1	—	0.500	<0.001
PaCO_2_ (mmHg)	0.268	<0.001	0.403	<0.001
HCO_3_^−^ (mmol/l)	0.280	<0.001	0.427	<0.001
Lac (mmol/l)	0.122	0.100	0.013	0.894
D-Dimer (*μ*g/ml)	0.318	<0.001	0.220	0.027

Abbreviations: PASP—pulmonary arterial systolic pressure; PaCO_2_—partial pressure of carbon dioxide; HCO_3_^−^—bicarbonate ion; NLR—neutrophil-to-lymphocyte ratio; PLR—platelet-to-lymphocyte ratio; SII—systemic-immune-inflammation index; PaCO_2_—partial pressure of carbon dioxide; HCO_3_^−^—bicarbonate ion; Lac—lactic acid.

**Table 5:** Univariate and multivariate analysis of the effects of the baseline parameters on PH.

Factors	Univariate analysis		Multivariate analysis	
	OR (95% CI)	*p* value	OR (95% CI)	*p* value
Lymphocyte (10^9^/l)	0.226 (0.114, 0.448)	<0.001	1.055 (0.273, 4.078)	0.938
NLR	1.173 (1.081, 1.273)	<0.001	1.161 (0.924, 1.458)	0.200
PLR	1.006 (1.003, 1.009)	<0.001	1.003 (0.993, 1.013)	0.564
SII	1.001 (1.000, 1.001)	<0.001	0.999 (0.998, 1.001)	0.256
NT-proBNP (pg/ml)	1.003 (1.001, 1.004)	<0.001	1.002 (1.001, 1.003)	<0.001
PaCO_2_ (mmHg)	1.047 (1.019, 1.075)	<0.001	1.018 (0.939, 1.104)	0.664
HCO_3_^−^ (mmol/l)	1.103 (1.042, 1.167)	<0.001	0.981 (0.822, 1.170)	0.828
Lac (mmol/l)	1.911 (1.130, 3.234)	0.016	1.663 (0.837, 3.305)	0.146
D-Dimer (*μ*g/ml)	1.910 (1.235, 2.953)	0.0036	1.581 (0.960, 2.603)	0.072

Abbreviations: PH—pulmonary hypertension; PaCO_2_—partial pressure of carbon dioxide; HCO_3_^−^—bicarbonate ion; NLR—neutrophil-to-lymphocyte ratio; PLR—platelet-to-lymphocyte ratio; SII—systemic-immune-inflammation index; Lac—lactic acid; CI—confidence intervals; OR—odds ratio.

**Table 6:** Comparison of the discriminative ability of NLR, PLR, SII, and NT-proBNP to predict PH.

Parameters	NLR	PLR	SII	NT-proBNP
Cut-off value	4.659	160.0	1012	384.0
AUC	0.701	0.669	0.670	0.776
95% CI	0.629, 0.766	0.596, 0.736	0.597, 0.737	0.709, 0.834
Sensitivity (%)	81.2	77.2	70.3	58.4
Specificity (%)	59.5	53.6	59.5	92.9
Positive predictive value (%)	70.7	66.7	67.6	90.8
Negative predictive value (%)	72.5	66.2	62.5	65.0
Accuracy (%)	71.4	66.5	65.4	74.1
Associated criterion	0.407	0.308	0.298	0.513
*N*	181	108	167	128

Abbreviations: NLR—neutrophil-to-lymphocyte ratio; PLR—platelet-to-lymphocyte ratio; SII—systemic-immune-inflammation index; AUC—area under the curve; CI—confidence interval.

## Data Availability

The data used to support the findings of this study are included within the supplementary information file.

## Supplementary Materials

Supplementary Materials Patients diagnosed with AECOPD (*n* = 185) were registered in this retrospective study. All patients evaluated for PH in our study underwent Doppler echocardiography and were divided into study and control groups depending on whether they also had PH. 101 AECOPD patients with PH were included in the PAH group, and the remaining eighty-four patients were assigned to the COPD group. Clinical characteristics and baseline laboratory tests (routine blood test (RBT), blood gas analysis, and amino terminal pro-B-type natriuretic peptide (NT-proBNP)) were tested at enrollment. All these data were listed in the supplementary file. There are some things particularly revelatory here: (1) in the gender column, 1 is for male and 2 is for female; (2) in the Respiratory failure, Hypertension, and Diabetes columns, 1 is for no and 2 is for yes; (3) BMI is defined as a person's weight in kilograms divided by the square of the height in meters (kg/m^2^); (4) the definition of the smoking index is the average root number per day multiplied by years of smoking; (5) inflammatory indices were calculated as follows: NLR = neutrophil counts/lymphocyte counts; PLR = platelet counts/lymphocyte counts; SII = platelet counts × neutrophil counts/lymphocyte counts; (6) abbreviations: LAD—left atrium diameter; LVDD—left ventricular end diastolic diameter; RAD—right atrium diameter; RVD—right ventricular diameter; PTRV—peak tricuspid regurgitation velocity; AECOPD—acute exacerbation of chronic obstructive pulmonary disease; PH—pulmonary hypertension; BMI—body mass index; WBC—white blood cell; RBC—red blood cell; NLR—neutrophil-to-lymphocyte ratio; PLR—platelet-to-lymphocyte ratio; SII—systemic-immune-inflammation index; PaCO_2_—partial pressure of carbon dioxide; HCO_3_^−^—bicarbonate ion; Lac—lactic acid; PASP—pulmonary arterial systolic pressure; PaCO_2_—partial pressure of carbon dioxide; HCO_3_^−^—bicarbonate ion; NLR—neutrophil-to-lymphocyte ratio; PLR—platelet-to-lymphocyte ratio; SII—systemic-immune-inflammation index.
